# Comparison of an Open versus List-Based Dietary Recall Method to Assess Unhealthy Feeding Practices among Infants and Young Children^☆^

**DOI:** 10.1016/j.cdnut.2024.104504

**Published:** 2024-11-19

**Authors:** Katelyn Yuen-Esco, Guy-Marino Hinnouho, Elaine L Ferguson, Ngik Rem, Hou Kroeun, Chea Mary, Alissa M Pries

**Affiliations:** 1 Helen Keller International, New York, NY, United States; 2 ICF International Inc, Rockville, MD, United States; 3 Department of Population Health, London School of Hygiene and Tropical Medicine, London, United Kingdom; 4 Ministry of Health, Phnom Penh, Cambodia

**Keywords:** unhealthy foods and beverages, dietary survey, infant and young nutrition, dietary assessment, infant and young child feeding, complementary feeding, diets, unhealthy foods

## Abstract

**Background:**

Collecting accurate dietary data is critical for assessing infant and young child feeding practices, identifying populations at risk, and using evidence to inform policy. In 2021, World Health Organization/United Nations Children’s Fund released new indicators of unhealthy food and beverage consumption and recommended that survey administrators use either an open or list-based method.

**Objectives:**

This study compared infants’ and young children’ unhealthy food consumption estimated using an open 24-h recall (24HR) compared with list-based 24HR among young children living in peri-urban Cambodia and explored the effect of social desirability bias on respondents’ responses.

**Methods:**

We conducted a secondary analysis of unhealthy food consumption estimated in a longitudinal cohort study implemented from June 2021 through January 2022 in the rural/peri-urban district of Khsach Kandal, Kandal Province, Cambodia (567 children aged 10–13.9 mo at baseline). Each month, for 5 mo, data were collected via an open 24HR. At the 6th month, half of the children were randomly assigned to also receive a list-based 24HR to collect data on unhealthy food consumption.

**Results:**

The prevalence of sweet beverage and unhealthy food consumption and zero fruit and vegetable consumption among young children was high. We observed that the percentage of children consuming sweet foods was significantly higher when estimated using the list-based compared with an open 24HR method (61.6% compared with 43.8%; *P* = 0.012). An association between social desirability bias and reported consumption of salty/fried foods was also observed across both groups; however, this relationship was more pronounced among caregivers who received the list-based 24HR than the open 24HR (*P* = 0.004).

**Conclusions:**

Researchers must carefully consider the method used for 24HR because this may have implications for respondents’ recall and memory. As more evidence is collected on the rising consumption of unhealthy food and beverages among young children, researchers should take into consideration the effects of caregiver’s susceptibility to social desirability bias when analyzing these consumption patterns.

## Introduction

Unhealthy foods, such as salty snacks, sugar-sweetened drinks, and confectionery items, are increasingly consumed around the world [1]. These foods are often nutrient-poor and high in salt, added sugar, and trans fats [1]. Although many of these foods are commercially produced, unhealthy foods can also be home prepared or sold by informal vendors [[2], [3], [4], [5], [6]].

For older infants and young children (IYC) aged 6–23 mo, high intakes of these foods can displace consumption of nutritious foods potentially leading to inadequate micronutrient intakes and poor growth [[7], [8], [9]]. Additionally, as early consumption of such foods has been associated with the establishment of long-lasting taste preferences [10,11], these consumption patterns carry increased risk of overweight and obesity later in life [12,13]. Increasingly, national dietary guidelines recommend avoidance of sugary beverages and ultraprocessed foods for children <2 y of age [[14], [15], [16], [17]]. However, there remains an evidence gap on consumption of unhealthy foods and sweet beverages during the complementary feeding period (age 6–23 mo), particularly in low- and middle-income countries (LMICs), and how this consumption changes as children age within the complementary feeding period [1,18,19].

In 2008, the WHO published a set of indicators for assessing IYC feeding practices, intended to support programmatic action and to contribute to monitoring global and national progress on feeding practices [20]. For over a decade, these indicators have been the global standard for data collection and reporting on IYC diets. Although this previous guidance was developed when undernutrition was a primary concern in many LMIC, current complementary feeding guidelines increasingly consider all forms of malnutrition including undernutrition, micronutrient deficiencies, and overweight or obesity [18]. Recognizing that the food landscape for IYC has evolved, the WHO and UNICEF published an updated set of indicators in 2021, which now also includes indicators of unhealthy food and beverage consumption [21].

To obtain data for these updated WHO/UNICEF indicators of unhealthy food consumption, several new questions were added to the recommended questionnaire assessing IYC diets, including whether children consumed any sweet foods or any salty/fried foods on the prior day. The 2021 WHO/UNICEF guide provides guidance to survey administrators on methods and notes that when asking about foods fed to the child, either an open or list-based 24-h dietary recall (24HR) method can be used [22]. In an open 24HR, the interviewer asks a series of standard probing questions to help the respondent recall all foods consumed in the previous day and night. In a list-based 24HR, the interviewer reads out a list of foods/groups to the respondent and the respondent indicates which were consumed during the specified recall period [22]. Each method has its advantages and disadvantages. With an open 24HR, the trained enumerator has the responsibility of matching foods recalled by the respondent to predetermined food groups. Conversely, for the list-based 24HR, this classification is left to the respondent, who may be less familiar with food categorization, and this could result in inconsistent reporting errors [23]. Although the open 24HR may be more intuitive, lead to a more complete recall of foods consumed and potentially a more accurate classification of foods into food groups than the list-based 24HR, it requires a longer training time for enumerators. It may also be more susceptible to recall bias, with respondents forgetting to mention foods fed in small amounts or outside of meals.

For both methods, the 24HR can be subject to systematic errors, including social desirability bias. Social desirability bias is the tendency for a respondent to exaggerate desirable qualities and minimize undesirable qualities [24]. There is evidence that social desirability bias may lead to misreporting of food intake [[25], [26], [27], [28]]. Although there is evidence of social desirability bias and an association with overreporting of foods considered to be healthy, it is plausible that social desirability bias may influence caregivers to underreport consumption of unhealthy foods [29].

Collecting accurate dietary data is critical for assessing IYC feeding practices nationally and globally, identifying specific populations at risk, and using evidence to inform nutrition policy decisions. To our knowledge, the comparability of list-based compared with open 24HR for measuring new WHO/UNICEF indicators of unhealthy diets among IYC has not yet been investigated. In this study, we aimed to address the evidence gap around these methodological questions for assessing unhealthy food/beverage consumption among IYC in an LMIC setting. The objectives of this study were to *1*) assess unhealthy food consumption practices each month across a 6-mo period among older IYC living in peri-urban Cambodia, *2*) compare prevalence of IYC unhealthy food consumption estimates from an open 24HR compared with list-based 24HR, and *3*) explore the effect of social desirability bias on respondents’ open and list-based 24HR responses.

## Methods

### Study setting, design, and participants

This study involved secondary analysis of data collected in a longitudinal cohort study implemented from June 2021 through January 2022 in the rural/peri-urban district of Khsach Kandal, Kandal Province, Cambodia. A detailed description of the longitudinal cohort study design has been published elsewhere [30]. Briefly, prior to data collection, a household census was conducted among the 93 villages of Khsach Kandal district, to enumerate all children within the 10–13.9 mo age range for enrollment. Because of an outbreak of COVID-19, the census was stopped after 66 of the 93 villages were completed, covering approximately two-thirds of the district’s population. All primary caregivers of eligible children who were identified during the census were contacted by telephone to be part of the study. Data were collected monthly for a 6-mo duration. Data collection was conducted via a telephone interview, to ensure participant safety during the COVID-19 pandemic. At each monthly timepoint, a structured questionnaire was first administered to the primary caregiver followed by a 24HR to assess their child’s food and beverage consumption in the previous day. All tools for this survey were translated into Khmer, back-translated to English to ensure accuracy, and pretested before data collection to ensure participant comprehension. Ethical approval for the study was provided by the Cambodian National Ethics Committee for Health Research and the London School of Hygiene and Tropical Medicine. Verbal informed consent was obtained from all primary caregivers prior to enrollment. Interviews lasted 15 min on average, and after each interview, caregivers were provided phone credit to compensate them for their time.

### Data collection

At each timepoint, an interviewer-administered questionnaire was used to collect information on demographic and socioeconomic characteristics pertaining to the household (asset ownership, housing materials, access to utilities, and food security) and to the child (sex, age, and breastfeeding status). At the final timepoint of data collection, when children were 15–18.9 mo of age, a measure of social desirability was collected, using a 13-question module adapted from Reynolds’ short forms of the Marlow-Crowne social desirability scale [31].

Following the questionnaire, caregivers were led through an open 24HR, where information on the types of foods and beverages consumed by the child during the previous day was collected. Caregivers were asked to recall all foods (dishes or single food items) consumed by the child on the day prior to interview, with probing used to facilitate caregiver’s recall of all items consumed including those between meals. After this recall, caregivers were asked to provide further details of each item, including ingredients in dishes and if foods/beverages were commercially produced. Standardized probes and prompts were used to obtain these details so that all information was captured consistently across interviewers. In cases where the child was fed by another caregiver and the primary caregiver did not know exactly what the child ate, this other caregiver was called with the help of the primary caregiver to recall what the child ate. Dietary data from the open 24HR were first collected on paper forms that were thoroughly reviewed by a study supervisor after each interview and then entered into the INDDEX24 mobile app on the Samsung tablet. Data on the paper forms and in the tablet were cross-checked by study supervisors to ensure data quality and then submitted to the INDDEX24 web app at the end of each day of data collection. The individual foods and beverages captured by the open 24HR were matched to food group categories later during analysis.

As a comparison group to the open 24HR method, caregivers of half the children at timepoint 6 (15.0–18.9 mo of age) were randomly assigned to receive a list-based 24HR where they were directly asked about their child’s consumption of sentinel sweet and salty/fried foods in the previous day. This comparison group is referred to as group A and this list-based 24HR was administered in the same interview before the open 24HR. Because the interviews were telephone-based because of COVID-19, and it was critical to minimize the length of the interview, only these 2 list-based questions were asked, rather than a full list of all food groups potentially consumed by the child. Sweet foods included candies, chocolate, and other sugar confections, including those made with real fruit or vegetables like candied fruit or fruit roll-ups, frozen treats like ice cream, gelato, sherbet, sorbet, popsicles or similar confections and cakes, pastries, sweet biscuits and other baked or fried confections which have at least a partial base of a refined grain, including those made with real fruit or vegetables or nuts, like apple cake or cherry pie. Salty or fried foods included chips, crisps, cheese puffs, French fries, fried dough, instant noodles, and similar items which contain mainly fat and carbohydrate and have at least a partial base of a refined grain or tuber. These sentinel unhealthy foods included commercially produced, vendor-prepared, and homemade foods. These questions for these categories of foods were based on the recently updated 2021 WHO infant and young child feeding indicator guidance [21] and included sentinel foods identified as commonly consumed by Cambodian IYC in prior research [32]. Group B was the other half of the sample that only received the open 24HR (*n* = 251), and consumption of these same sweet and salty/fried foods was identified through their open 24HR responses.

Interviews were conducted by 9 enumerators from health/nutrition sciences background, who were trained over 2 weeks on the survey methodology and tools. Training involved classroom-based instruction and practice in phone interviewing skills. Data from the practice sessions were checked daily and enumerators were retrained until they were proficient and comfortable with the survey questionnaires.

### Data analysis

All analyses were carried out using Stata 15 (StataCorp). The characteristics of the participating children and households were presented as means ± SD or proportions as appropriate. Differences in sociodemographic characteristics were tested between groups A and B using chi-squared tests. Food security was assessed using the Household Food Insecurity Access Scale and households were categorized as food secure or food insecure (mild/moderate/severe) [33].

Data from the open 24HR were used to calculate 3 WHO/UNICEF indicators of unhealthy diets: *1*) proportion of children consuming zero vegetable or fruit, *2*) proportion of children consuming sweet beverages, and *3*) proportion of children consuming unhealthy foods [22]. “Sweet beverages” included soft drinks, fruit-flavored drinks, 100% fruit juice, sports drinks, energy drinks, chocolate or malt-flavored drinks, sweetened milk drinks, and included commercially produced drinks, vendor-prepared, and homemade drinks. The proportion of children for each of these 3 indicators was calculated at all timepoints; however, group A was excluded from prevalence estimates at timepoint 6 because receiving the list-based 24HR for unhealthy food categories (sweet foods and salty/fried foods) could have biased their recall during the open 24HR, making this timepoint incomparable to the previous 5 timepoints.

Comparison of the open 24HR and list-based 24HR methods was done at timepoint 6 by comparing consumption prevalence of the 2 unhealthy food categories (sweet foods and salty/fried foods) among the group A sample that received the list-based 24HR method compared with the prevalence among the group B sample that only received the open 24HR method. The proportions of children who consumed any sweet foods or salty/fried foods were calculated for group A and group B and compared using a chi-square test. Additionally, the agreement between the 2 methods of recall for participants in group A, who received both the list-based and open 24HR, was estimated using Cohen’s kappa [34].

A social desirability score was generated based on the sum of socially desirable answers out of the 13-question module; a score of 13 was the highest social desirability score. Logistic regression was used to assess whether reported consumption of unhealthy foods varied by levels of social desirability score.

## Results

A total of 567 caregivers of children aged 10–13.9 mo were enrolled and interviewed at timepoint 1. A total of 549, 539, 527, 523, and 501 caregivers were interviewed each subsequent month (timepoints 2–6), respectively. Sociodemographic characteristics for children at timepoint 6 and their households are summarized in Table 1. At timepoint 6, children were on average 17.4 mo old and 34.9% were still breastfeeding, with breastfed children receiving a median of 10 breastfeeds on the day before the interview. Approximately one-fifth (18.8%) of households were food secure.

**TABLE 1:** Sociodemographic characteristics of children and households at Timepoint 6.

	All (*n* = 501)	Group A: list-based recall (*n* = 250)	Group B: open recall only (*n* = 251)	*P* value^1^
Children				
Age (mo) mean ± SD	17.4 ± 1.1	17.4 ± 1.1	17.3 ± 1.1	0.987
Female sex, *n* (%)	249 (49.7)	114 (45.6)	135 (53.8)	0.067
Currently breastfed, *n* (%)	175 (34.9)	89 (35.6)	86 (34.3)	0.754
Morbidity in last 2 weeks, *n* (%)	319 (63.6)	155 (62.0)	164 (65.3)	0.437
Households				
Food security, *n* (%)				
Food secure	94 (18.8)	40 (16.0)	54 (21.5)	0.216
Mildly food insecure	68 (13.6)	30 (12.0)	38 (15.1)	
Moderately food insecure	327 (65.3)	173 (69.2)	154 (61.4)	
Severely food insecure	12 (2.4)	7 (2.8)	5 (2.0)	

1 On the basis of the chi-squared tests.

### Select infant and young child feeding practices

**FIGURE 1:**
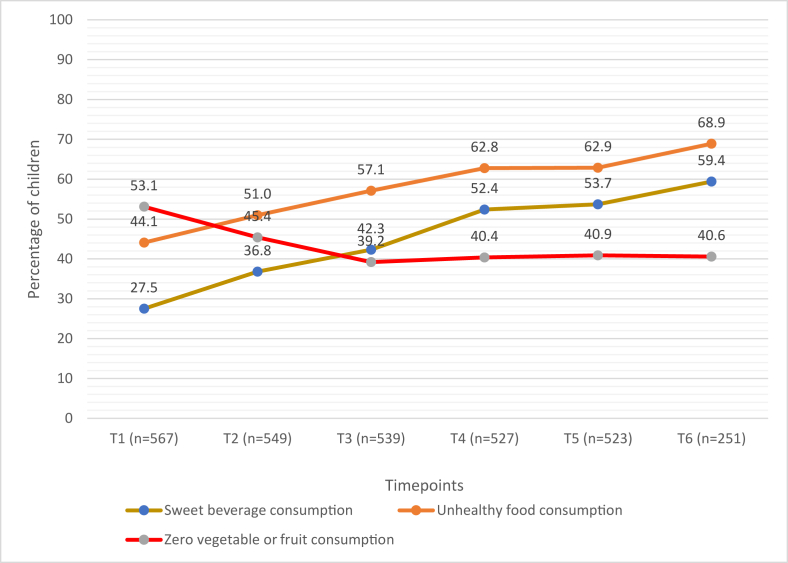
Percentage of children who consumed sweet beverage, unhealthy food, and zero fruit or vegetable across timepoints.

Figure 1 shows the percentage of children with selected unhealthy infant and young child feeding practices across timepoints. Zero consumption of vegetables or fruits decreased with child age, whereas consumption of unhealthy foods and sweet beverages increased steadily with child age. By 15.0–18.9 mo of age, the majority of children had consumed unhealthy foods and sweet beverages the previous day (68.9% and 59.4%, respectively).

The most commonly consumed unhealthy food and beverage items among children 10.0–18.9 mo old are presented in Table 2. The most commonly consumed sweet food across all timepoints was cake. At timepoint 1, 18.9% of children 10.0–13.9 mo of age had consumed cake in the previous day, and this percentage increased to 25.5% at timepoint 6 when children were 15.0–18.9 mo of age. Among the unhealthy salty/fried foods, salty crackers were the most commonly consumed item—over a third of children across the last 3 timepoints were consuming this food. At timepoint 1, 18.7% of children 10.0–13.9 mo old had consumed sweetened cow milk in the previous day; this percentage more than doubled by timepoint 6, with 51.0% of children 15.0–18.9 mo old consuming this type of sweet beverage in the previous day. Although the consumption of other sweet beverages was substantially lower, several children consumed energy drinks, reaching 4.6% of 14.0–17.9 mo olds at timepoint 5, as well as 4.8% of children 15.0–18.9 mo of age consuming a soft drink. The unhealthy foods consumed by the children were predominantly commercially produced, accounting for 78.8% of unhealthy foods at 10.0–13.9 mo of age to 90.9% of unhealthy foods at 14.0–17.9 mo of age.

**TABLE 2:** Most commonly consumed unhealthy food and beverage items among children 10.0–17.9 mo old at 5 monthly timepoints^1^.

Items consumed	T1 10.0–13.9 mo (*n* = 567)	T2 11.0–14.9 mo (*n* = 549)	T3 12.0–15.9 mo (*n* = 539)	T4 13.0–16.9 mo (*n* = 527)	T5 14.0–17.9 mo (*n* = 523)	T6 15.0–18.9 mo (*n* = 251)
Unhealthy sweet foods						
Cake	107 (18.9)	118 (21.5)	138 (25.6)	144 (27.3)	138 (26.4)	64 (25.5)
Dessert soup	23 (4.1)	31 (5.7)	19 (3.5)	32 (6.2)	34 (6.5)	20 (8.0)
Sweet bread	22 (3.9)	24 (4.4)	25 (4.6)	36 (6.8)	43 (8.2)	24 (9.6)
Jelly candy	11 (1.9)	14 (2.6)	8 (1.5)	12 (2.3)	14 (2.7)	13 (5.2)
Unhealthy salty/fried foods						
Salty crackers	115 (20.3)	143 (26.1)	171 (31.7)	180 (34.2)	182 (34.8)	85 (33.9)
Fried cassava balls	9 (1.6)	13 (2.4)	14 (2.6)	24 (4.6)	25 (4.8)	15 (6.0)
Instant noodles	8 (1.4)	12 (2.2)	16 (3.0)	10 (1.9)	20 (3.8)	14 (5.6)
Deep fried banana	6 (1.1)	4 (0.7)	6 (1.1)	7 (1.3)	7 (1.3)	1 (0.4)
Sweet beverages						
Sweetened cow milk	106 (18.7)	148 (27.0)	185 (34.3)	214 (40.6)	219 (41.9)	128 (51.0)
Packaged tea drink	18 (3.2)	17 (3.1)	19 (3.5)	18 (3.4)	26(5.0)	10 (4.0)
Sweetened soy milk	15 (2.7)	22 (4.0)	13 (2.4)	18 (3.4)	20 (3.8)	7 (2.8)
Energy drink	9 (1.6)	12 (2.2)	12 (2.2)	20 (3.8)	24 (4.6)	7 (2.8)
Juice drink	8 (1.4)	12 (2.2)	12 (2.2)	16 (3.0)	19 (3.8)	12 (4.8)
Soft drink	6 (1.1)	10 (1.8)	5 (0.9)	8 (1.5)	16 (3.1%)	3 (1.2)

Abbreviation: T, timepoint.

1 Values presented as *n* (% consumers).

### Comparison and agreement between the 2 methods for measuring the consumption of unhealthy sweet foods and salty/fried foods

**FIGURE 2:**
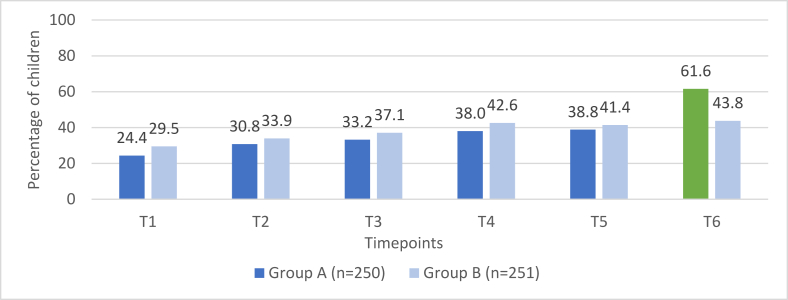
Consumption of sweet foods among group A compared with group B.

**FIGURE 3:**
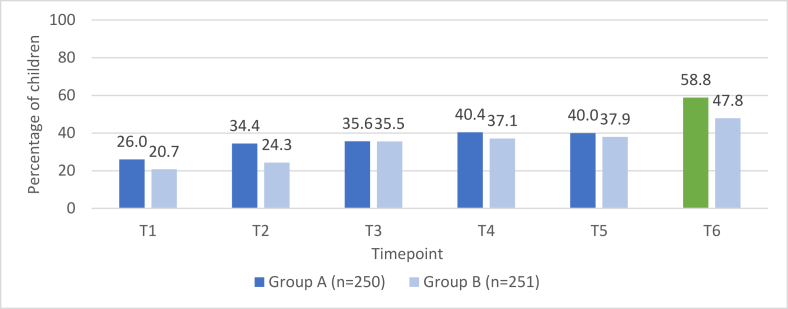
Consumption of salty/fried foods among group A compared with group B.

Prevalence of consumption of sweet foods and salty/fried foods among group A (*n* = 250) compared with group B (*n* = 251) across the 6 timepoints are presented in FIGURE 2, FIGURE 3. For timepoints 1–5, the prevalence rates for both groups are based on responses provided during the open recalls; the proportions of children consuming these food groups are similar across all timepoints, with the exception of salty/fried food consumption at timepoint 2 (*P* = 0.013). At timepoint 6, the prevalence rate presented for group A is from the list-based recall and is compared to the prevalence rate for group B from the open recall. The proportions of children consuming sweet foods and salty/fried foods were higher among group A (list-based recall) as compared with group B (open recall) with this difference statistically significant for sweet foods: 61.6% compared with 43.8% (*P* = 0.012) and 58.8% compared with 47.8% (*P* = 0.082), respectively.

Within group A, agreements between the responses from the list-based 24HR compared with responses from the open 24HR were high for both sweet food consumption and salty/fried food consumption on the previous day (proportion of agreement = 93.6%; kappa =0.866) and (proportion of agreement = 93.6%; kappa = 0.869), respectively. For sweet foods, consumption was reported by 61.6% (*n* = 154) and 60.0% (*n* = 150) of respondents when receiving the list-based and open recall, respectively. For salty/fried foods, consumption was reported by 58.8% (*n* = 147) and 56.4% (*n* = 141) of respondents when receiving the list-based and open recall, respectively.

### Social desirability

At timepoint 6, there was evidence of a relationship between social desirability bias and reported consumption of salty/fried foods in group A when receiving the list-based recall, but not for reported consumption of sweet foods (Table 3). Among group A caregivers who were administered the open 24HR after receiving the list-based 24HR on unhealthy food consumption, a 1-point higher social desirability score was significantly associated with an 18.9% lower odds of salty/fried food consumption. Among the group B caregivers who only received the open 24HR, there was a marginal association between higher social desirability score and lower reported consumption of salty/fried foods by caregivers.

**TABLE 3:** Association between social desirability score^1^ and consumption of sweet or salty/fried foods by group A [list-based 24-h recall (24HR)] and group B (24HR).

List-based 24HR (*n* = 250)	Social desirability score			
	*N* = 250	Mean ± SD	*β* (95% CI)	*P*
Sweet foods consumption				
No	96	7.6 ± 1.9		
Yes	154	7.7 ± 1.8	0.01 (–0.13, 0.15)	0.923
Salty/fried foods consumption				
No	103	8.1 ± 1.9		
Yes	147	7.4 ± 1.7	–0.21 (–0.36, –0.07)	0.004
Open recall 24HR only (*n* = 251)	*N* = 251	Mean ± SD	*β* (95% CI)	*P*
Sweet foods consumption				
No	141	7.9 ± 1.9		0.132
Yes	110	7.6 ± 1.7	–0.11 (–0.25, 0.03)	
Salty/fried foods consumption				
No	131	8.0 ± 1.7		
Yes	120	7.5 ± 1.8	–0.11 (–0.25, 0.03)	0.069

1 Higher social desirability scores indicate greater levels of respondent bias toward statements of social approval.

## Discussion

This study in rural/peri-urban Cambodia found a high prevalence of sweet beverage and unhealthy food consumption among children under 2 y of age when using the new WHO indicators, with most of these unhealthy beverages and foods being commercially produced. Furthermore, a concerning proportion of children consumed zero fruits or vegetables across the complementary feeding period. Differences in consumption rates for unhealthy foods were observed between groups that received a list-based 24HR compared with an open 24HR, indicating that the type of method used for assessing unhealthy food consumption may have implications for respondents’ recall. An association between social desirability bias and reported consumption of salty/fried foods was also observed across both groups; however, this relationship was more pronounced among caregivers who received the list-based 24HR. Our study highlights several methodological issues for researchers to consider when assessing consumption of unhealthy foods and beverages using the new WHO indicators.

The high prevalence of unhealthy food and sweet beverage consumption observed in this study among older IYC in rural/peri-urban Cambodia is consistent with other research conducted in the Southeast Asia region. A recent study in Thailand measuring the newly updated WHO complementary feeding indicators among a nationally representative sample of IYC found that 72.2% and 74.0% of 12.0–17.0-mo olds had consumed unhealthy food and sweet beverages, respectively, in the previous day [35] compared with 62.9% and 53.7%, respectively, of children aged 14.0–17.9 mo in our cohort. However, our finding that 40.9% of children aged 14.0–17.9 mo in Cambodia did not consume any vegetables or fruit in the previous day is substantially higher than the 13.9% observed in 2022 among Thai children 12.0–17.0 mo of age [35]. Low consumption of vegetables and fruit is associated with increased risk of noncommunicable diseases and low intake of fruits has been shown to be a leading dietary risk factor for mortality in later life [36,37]. Prior research shows that consumption of unhealthy foods early in life establishes long-lasting taste preferences [10,11], and there is some evidence that low fruit and vegetable intake during infancy is associated with low intakes later in childhood [38]. Further attention should be paid to the high consumption of unhealthy food and sweet beverages in early childhood in this region. More research is needed to understand why an alarming proportion of young children in Cambodia are not consuming enough vegetables and fruits and young child feeding interventions should be designed to intervene early in life.

To the best of our knowledge, this is the first study to compare a list-based 24HR method with an open 24HR method for measuring the new WHO/UNICEF indicators of unhealthy diets among IYC. Although researchers and national population-based surveys have begun measuring WHO/UNICEF indicators of unhealthy diets among IYC, there is a lack of evidence on the measurement implications of administering a list-based compared with an open 24HR. This present study among IYC in rural/peri-urban Cambodia found that reported consumption of sweet foods and salty/fried foods was higher among respondents who received a list-based 24HR compared with those who only received an open 24HR. In prior timepoints, prevalence rates for unhealthy food consumption based on open 24HR were similar between the 2 comparison groups, indicating that the difference noted at timepoint 6 was likely because of differential reporting between the list-based compared with open-recall methods. Additionally, kappa statistics indicated strong agreement of reported consumption rates based on the list-based recall and open recall among group A caregivers who received both methods. When considered together, these findings indicate that the percentage of children consuming unhealthy foods might be higher when estimated using a list-based 24HR method compared with an open-recall 24HR. This could be because of a list-based 24HR method improving respondents’ memory and recall. The role of recall bias in open-recall 24HR has been previously noted, with prior studies noting snack foods to be particularly susceptible to omissions in participants’ memory of consumption. A study among adults in New Zealand compared consumption results from an open-recall 24HR versus by a wearable camera and found underreporting most commonly occurred for foods consumed as snacks, including unhealthy snacks such as biscuits and healthy snacks such as fruit [39]. A recent study examining the use of pictorial recall aids to reduce recall bias in quantitative 24HR in urban Nepal and peri-urban Senegal found that unhealthy snack foods, such as biscuits and chips, were among the foods most omitted by respondents during open recalls [40]. Prior research comparing dietary diversity indicators assessed using a list-based compared with open 24HR is limited and shows conflicting results. One study found the agreement between a list-based and open 24HR assessment of minimum dietary diversity for women was lower in India compared with Bangladesh [41]. In a recent study conducted among IYC in Cambodia and Zambia, researchers found that administering the list-based recall and the multiple-pass open recall were both equivalent to in-home observation when measuring minimum dietary diversity in the Cambodia setting. However, they both significantly overestimated the prevalence of minimum dietary diversity in Zambia, because of an overestimation of foods from the flesh foods, eggs, and vitamin A-rich fruits and vegetables food groups [42]. The nature of diets and respondent comprehension can differ across contexts and further research in other cultural contexts is needed to evaluate the predictive performance of list-based 24HR and open 24HR in measuring various nutrition indicators.

A negative association was found between social desirability score and caregivers’ reported consumption of salty/fried food among IYC, particularly when caregivers were directly asked about their child’s consumption of these foods in a list-based recall. This finding indicates that caregivers who were concerned with providing socially acceptable responses may be less likely to report their child’s consumption of foods that are perceived as unhealthy. A study in the United States among caregivers of children 24–60-mo olds reported similar results, where higher social desirability scores were found among caregivers who reported low fast food and snack consumption compared with caregivers who reported high fast food and snack consumption [29]. It is possible that some caregivers may feel ashamed to report that they have been feeding their young child unhealthy foods that are high in salt and unhealthy fats. This explanation is consistent with prior research showing that social desirability is more strongly associated with negative parenting behaviors compared with positive parenting behaviors [43]. Although our study did not find an association between social desirability and reported consumption of sweet foods, other studies suggest that some caregivers may selectively underreport their children’s consumption of unhealthy items such as sugary foods and sugar-sweetened beverages [25,44].

The presence of social desirability bias within dietary assessments has been shown in prior studies. In a randomized blinded trial comparing a food frequency questionnaire to an open 24HR in the measurement of self-reported fruit and vegetable consumption, both methods were found to be susceptible to substantial social desirability bias [28]. The study also found that half of the participants who were randomly selected to receive a letter prior to the interview describing the investigation as a study of fruit and vegetable intake reported consuming more fruits and vegetables than those who were only told the study was on general nutrition. Our study suggests that social desirability bias may also result in respondents’ underreporting of unhealthy dietary practices in this Southeast Asian context. These findings have implications for nutrition researchers and survey implementers. Researchers should consider that social desirability may affect caregiver reports of their children’s unhealthy food intake and address this through measurement of social desirability bias during data collection and adjustment of results where an association is detected. Further research is needed to investigate potential strategies to limit social desirability bias in surveys, such as training data collectors to establish rapport between respondents and to detect social desirability based on behavioral cues [45].

The primary limitation of our study was the lack of a “gold standard” dietary intake measure, such as direct observation, that does not rely on recalls to compare the self-reported dietary assessments in the list-based and open 24HR. Without an objective measure, it is not possible to determine if the list-based 24HR overreported or the open 24HR underreported unhealthy food consumption in this context. Objective measures of dietary intake such as weighed food records or detailed observations are often burdensome [42,46,47] and were not possible to conduct within this study because of logistical challenges and the outbreak of COVID-19. Although findings from this study indicate that the method used to measure consumption of unhealthy foods may impact results, with list-based 24HR yielding higher consumption prevalence estimates than the open 24HR, validation studies are needed. Additionally, this study only used 2 questions in the list-based 24HR, instead of the full list of food category questions outlined in the WHO IYCF indicator questionnaire. This approach was taken because of the COVID-19 outbreak which required a telephone-based survey. Using the full list would have required substantially more time, increasing respondent burden and potentially resulting in respondent fatigue, as evidenced in other studies utilizing telephone-based surveys [48,49]. However, using the full list could have led to different results, and additional studies comparing the full list-based 24HR with an open 24HR are warranted. Similarly, further research comparing the survey mode effects of telephone-based surveys compared with face-to-face surveys would also be of value. Finally, although in-person data collection was not possible because of COVID-19, the use of a phone-based interview could be a limitation of this study.

This is one of few studies that have utilized the newly updated WHO indicators of unhealthy feeding practices among older IYC in the Southeast Asia region. The high prevalence of unhealthy food, sweet beverage, and no fruit or vegetable consumption noted among children under 2 y of age in rural/peri-urban Cambodia highlights the importance of measuring unhealthy diet indicators for this young age group and the urgent need to address suboptimal complementary feeding practices in the region. The differences in consumption prevalence rates noted between groups that did or did not receive the list-based 24HR suggest that survey designers and researchers must carefully consider the method used for dietary assessments, as this may have implications for respondents’ recall and memory. In trying to understand discrepancies between reported consumption of unhealthy foods, specifically salty/fried foods, social desirability bias may account for some of the variance. Caregivers may feel ashamed that they feed their children salty/fried foods and consequently underreport this practice. In some contexts, researchers should consider the effects of caregivers’ susceptibility to social desirability bias when analyzing patterns of unhealthy food consumption.

## Author contributions

The authors’ responsibilities were as follows – AMP, G-MH, ELF: designed the research; AMP, ELF, G-MH, HK, CM: wrote the study protocol; NR, G-MH: conducted the research; G-MH: analyzed the data; KY-E, AMP, G-MH: wrote the paper; AMP, KY-E: had primary responsibility for final content; and all authors: read and approved the final manuscript.

## Conflict of interest

The authors report no conflicts of interest.

## Funding

This work was supported, in whole or in part, by the Bill & Melinda Gates Foundation [OPP1190179]. Under the grant conditions of the Foundation, a Creative Commons Attribution 4.0 Generic License has already been assigned to the Author Accepted Manuscript version that might arise from this submission. The supporting source had no involvement or restrictions regarding submission for publication.

## Data availability

Data described in the manuscript, code book, and analytic code will be made available upon request pending approval by Helen Keller International. The data used for this study can be accessed by contacting Helen Keller Intl at data@hki.org.
